# Shear Capacity of RC Beams Strengthened with Flax Fiber Sheets Grafted with Nano-TiO_2_

**DOI:** 10.3390/ma13061430

**Published:** 2020-03-20

**Authors:** Hongguang Wang, Guijun Xian

**Affiliations:** 1 School of Civil Engineering, Northeast Forestry University, Harbin 150040, China; wanghongguang@nefu.edu.cn; 2 School of Civil Engineering, Harbin Institute of Technology, Harbin 150090, China

**Keywords:** RC beam, flax fiber sheets, nano-TiO_2_, shear strengthening

## Abstract

Flax fiber sheets provide the advantages of high specific strength, a short growth cycle, environmental friendliness, wide availability, and low cost. Therefore, in this study, the shear capacities of reinforced concrete (RC) beams strengthened with ordinary flax fiber sheets, flax fiber sheets grafted with nano-TiO_2_, and unidirectional basalt fiber sheets were compared. The bearing characteristics and failure modes of RC beams strengthened with flax fiber sheets were investigated. The results showed that after reinforcement with flax fiber sheets, the bearing capacity and mid-span deflection of the RC beams are considerably improved, and the reinforcing effect of flax fiber sheets grafted with nano-TiO_2_ is greater than that of unmodified flax fiber sheets. After reinforcement with flax fiber sheets grafted with nano-TiO_2_, the shear capacity of the RC beams is considerably improved to 653 kN, which is 72.8% higher than that of the unreinforced RC beams. Meanwhile, the mid-range deflection of the beam reached 14.6 mm, which is 75.9% higher than that of the unreinforced RC beams.

## 1. Introduction

China is the world’s largest consumer of concrete. Under the dual action of long-term exposure to the natural environment and service loads, the bearing capacity of concrete structures gradually decreases [1]. Therefore, the reliability of the structure will gradually decline, endangering the safe use of the structure [2,3]. With the development of urbanization and the national economy in China, a huge market demand is expected in the field of building maintenance and reinforcement. If this is not adequately anticipated and if corresponding technical measures are not adopted for the projects, the country will not be able to afford such huge maintenance and reinforcement costs in the coming decades. In recent years, the application of fiber reinforced plastic (FRP) composite materials for civil engineering reinforcement has been a research hotspot in China and across the globe [4,5,6,7]. FRP composites are a type of composite material composed of a polymer matrix and fiber material; these materials can be produced by spraying, molding, pultrusion, winding, and other forming processes [8,9,10].

The mechanical and physical properties of the natural fibers are dependent on the chemical compositions of the fibers. The major components of natural fibers are cellulose, hemi-cellulose and pectin. Research has indicated that flax fiber sheets and their corresponding composites have mechanical properties similar to those of glass fiber composites [11,12,13,14]. It can thus fully satisfy the requirements for the capacity of structural materials in civil engineering [15]. The unique mechanical properties of natural fiber composite make its application in civil engineering full of unknowns and challenges. The grafting of selected chemicals onto the natural fiber surfaces enhanced the mechanical properties and the bonding strengths of various natural fibers to resin matrices [15]. The grafting of nano-TiO_2_ onto the surface of flax fiber sheets can not only change the chemical composition and surface morphology of the flax fiber sheets, but it can also transform the hydrophilic surface into a lipophilic surface, which improves the compatibility of the flax fiber monofilament with epoxy resin [15,16]. After grafting, a nanoparticle layer structure is produced on the surface of the flax fiber. This can create new covalent bonds, such as -Ti-O-Si- and -Si-O-C-. The degree of defects on the surface of the flax fiber sheets is reduced, and a nano-reinforced epoxy resin composite layer is formed between the flax fiber sheets and the epoxy resin, which fully exerts the reinforcing effect of the nano-TiO_2_ [15,17].

In this study, the shear capacity, bearing characteristics, and failure modes of reinforced concrete (RC) beams and RC beams strengthened with ordinary flax fiber sheets, unidirectional basalt fiber sheets, and flax fiber sheets grafted with nano-TiO_2_ were analyzed and compared. An equation for the shear bearing capacity of RC beams strengthened with flax fiber sheets was also proposed.

## 2. Materials and Methods

### 2.1. Materials and Reagents

The RC beam was cast with commercial concrete. The concrete grade was C30, water–cement ratio was 0.42, and ratio of cement:water:sand:gravel was 360:151:632:1282 by weight. The cement was Swan brand P.O grade 42.5 ordinary Portland cement, produced by the Harbin Cement Factory of the Jilin Yatai Group, China. The maximum particle size of the coarse aggregate was approximately 25 mm. The measured concrete slump was 43 mm [18]. After pouring was completed, the RC beam was continuously watered at room temperature for 7 days, after which it was removed from the mold and rinsed until 28 days. According to the requirements in the standards for the test of mechanical properties of ordinary concrete (GB/T 50081-2011) [18], the compressive strength of the concrete cube (100 mm × 100 mm) was measured to be 38.2 ± 0.3 MPa.

The vertical, structural, and erecting steel rebars were HRB400 grade ordinary hot rolled ribbed steel bars of 25 mm, 14 mm, and 12 mm, respectively. The diameter of the stirrup was 8 mm, and was HPB300 grade hot rolled round bar. According to the requirements of the standard test method for tensile testing of metallic materials (GB/T 228-2010) [19], the mechanical properties of the steel bars were measured and are listed in Table 1.

**Figure 1:**
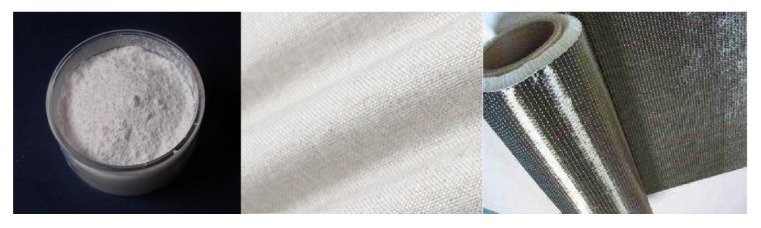
Photos for the nano-TiO_2_, the flax fiber sheets grafted with nano-TiO_2_, and the basalt fiber sheets.

To investigate the effect of flax fiber sheets on the shear resistance of RC beams at different directions and compositions, a flax fiber composite material was prepared using two-way linen fiber sheets produced by the China Harbin Flax Textile Co., Ltd. The density of the fiber cloth was 1.5 g/cm^3^, nominal thickness was 0.16 mm, and the spinning process was ring spinning. To compare the effects of different types of flax fiber sheets on the shear reinforcement of RC beams, basalt fiber sheets were also used to reinforce the RC beams in this study. The basalt fiber sheets were produced by the Sichuan Aerospace Tuoxin Basalt Industrial Co., Ltd. Chengdu, China. The main technical parameters of the nano-TiO_2_ (from Zhejiang Hongsheng Material Technology Co., Ltd. Zhoushan, China), the flax fiber sheets grafted with nano-TiO_2_ [15] and basalt fiber sheets [20] are summarized in Table 2, Table 3 and Table 4. The photos for the nano-TiO_2_, the flax fiber sheets grafted with nano-TiO_2_, and the basalt fiber sheets are given in Figure 1.

The epoxy resin (JZ-A) and curing agent (JZ-B) (purchased from Nan Ya Plastic Co.) were mixed at a mass ratio of 100:34.5 and cured at room temperature. According to ASTM D638 (standard test method for tensile properties of plastics), the tensile strength and modulus of the epoxy resin were measured in the laboratory, which were 59.1 MPa and 3.08 GPa, respectively. DMTA (dynamic mechanical thermal analysis) of the epoxy resin was performed by a dynamic mechanical thermal analysis machine (Q800, TA Instruments Co., New Castle, DE, USA), and the glass transition temperature was approximately 62.5 °C.

### 2.2. Instruments and Equipment

For testing the shear capacity of RC beams strengthened with flax fiber sheets, the main test instruments and equipment used are described in Table 5.

### 2.3. Characterization

#### 2.3.1. Structural Design of RC Beams

**Figure 2:**
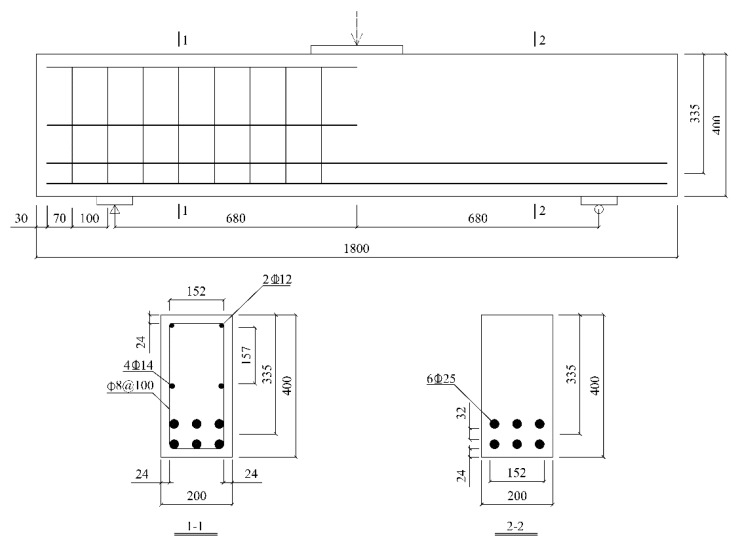
Main structural dimensions of RC beam (unit: mm).

Based on the tests of flax fiber sheet composites, the group of composite materials with excellent mechanical properties, thermal properties, and interface properties was selected for the shearing reinforcement of RC beams, and the performance of beams reinforced with ordinary flax fiber sheets was compared with that of beams reinforced with flax fiber sheets grafted with nano-TiO_2_. The RC beams have a length of 1800 mm and a section size of 200 × 400 mm. The main structural dimensions are shown in Figure 2.

According to the requirements in the Concrete Structure Design Code (GB 50010-2010), to produce shear failure of RC beams, a sufficient number of longitudinal steel bars should be placed in the span of the beams to ensure that bending failure does not occur before the shear failure. As shown in Figure 2, the RC beams are equipped with a certain number of stirrups in the left half-span, and half-span structural steel bars are arranged in the middle of the cross section to improve the shear capacity of the left half-span. This also ensures that the shear failure occurs only in the right-half span to facilitate the test measurements.

#### 2.3.2. FRP Strengthened RC Beams in Shear Capacity

In this study, the RC-control beam is not reinforced. RC-FRP1 and RC-FRP3 are reinforced with 3 and 6 layers of ordinary flax fiber sheets, respectively, and the flax fiber sheets in these beams are in the warp and weft directions, respectively. RC-FRP5 is reinforced with three layers of flax fiber sheets grafted with nano-TiO_2_; the flax fiber sheets are in the warp direction. The configuration of the reinforcement with fiber sheets is summarized in Table 6.

**Figure 3:**
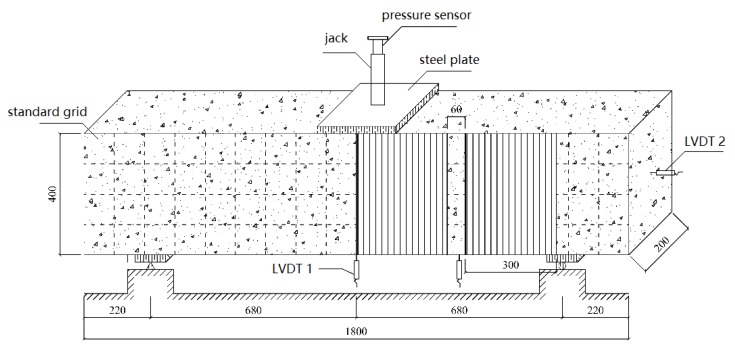
FRP strengthened RC beams program (unit: mm).

The width and length of the fiber sheets is 300 mm and 1000 mm, respectively. They are pasted as a U-shaped reinforcement, and the paste positioning is shown in Figure 3. To facilitate the centering and crack width measurement of the concrete beam in the loading device and description of test phenomena, the side of the RC-control beam is painted, and the extension direction is divided into a standard grid of 100 mm × 100 mm. During the test, a three-point bending load is applied and loaded by force (capacity of pressure sensor is 2500 kN). The size of steel plate is 200 × 200 mm. The support mode is a left fixed-hinge support and a right movable-hinge support. Linear variable differential transformer (LVDT, gauge length is 25 mm) sensors are arranged in the span of the RC beam, on the side of the bonded fiber sheets, and at the fulcrum to record the longitudinal displacement of the beam at different positions. A resistance strain gauge is attached to the concrete surface of the RC-control beam and the fiber sheet surface of the remaining beams to measure the strain generated by the concrete and the fiber sheets. In this study, the displacement and strain signals in the tests are converted into potential signals by a strain collector.

## 3. Results and Discussions

### 3.1. Experimental Results and Analysis of the Shear Capacity of Ordinary RC Beams

**Figure 4:**
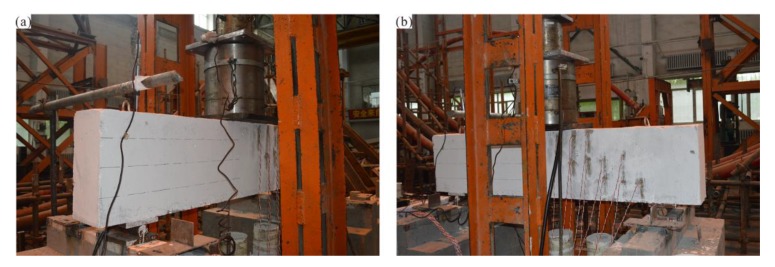
Test set-up on RC-control beam.

After 28 days of standard maintenance for all of the reinforced concrete beams, they were transferred to the test site and the fiber cloth was attached according to the experimental program for evaluating the shear capacity of FRP-strengthened RC beams. First, the shear capacity test is performed with the RC-control beam. Before the test, the side of the RC-control beam is painted to facilitate its centering on the loading device and the measurement or crack widths and test phenomena; the extension direction is divided into a 100 × 100 mm standard grid. The test set-up on the RC-control beam is shown in Figure 4.

At the beginning of the test, vertical micro-cracks appear near the beam span of the RC-control beam. There are also numerous tiny flexural cracks of the concrete beam in the unformed stirrups on the right side. As the load continues to increase, the number of cracks in the right half-span of the beam gradually increases and the cracks extend upward. The diagonal shear crack-based energy release is not significantly affected by the type of shear reinforcing bars, which are included the flexural shear cracks and web shear cracks. Stirrups often act as crack initiators and thus affect the flexural shear crack; hence, smaller flexural shear cracks would be formed in the case of closer stirrup spacing [21]. The web shear cracks could take place outside the zone affected by the support reaction. The traditional design method for web shear failure was included in Eurocode 2, and the model for shear compression failure is completely different from web shear failure and over-conservative [22].

**Figure 5:**
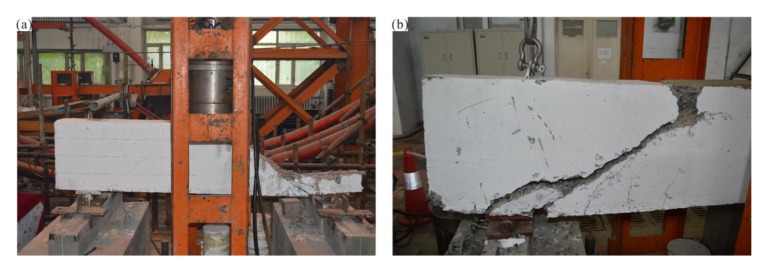
Failure mode and crack distribution of RC-con beam.

RC-control exhibits a typical shear failure mode, and its failure mode and crack distribution are shown in Figure 5. It can be clearly seen that one of the many shear cracks have the largest width and gradually forms a critical shear crack. After the appearance of critical shear cracks, the number and width of vertical cracks at the bottom of the mid-span section tends to stabilize. When the shear crack expands to a certain extent, it begins to branch near the bottom side of the beam, i.e., a gentle steel bond crack occurs. As the load increases, the critical shear crack continues to develop upward and extends below the mid-span loading point. At this point, no new shear cracks will occur until the concrete in the span is crushed and destroyed.

**Figure 6:**
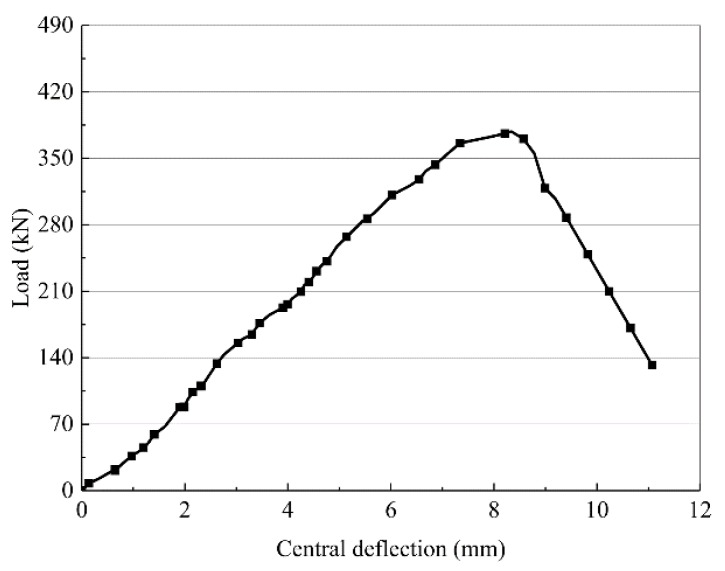
Central deflection and load curves of RC-con beam.

The central deflection and load curves for the RC-control beam are shown in Figure 6. The central deflection and load curves for RC-control can be divided into three main stress processes: the elastic phase, cracked working phase, and critical failure phase. At the beginning of loading, as the load is increased gradually, the mid-span deflection of the RC-control beam increases linearly. The flexural cracks gradually appear at the bottom of the beam [23]. When the load reaches approximately 176 kN (46.6%), a crack with the largest width can be identified in the right half-span of the beam, and a critical shear crack is gradually formed. When the load reaches 352 kN (93.1%), the critical shear crack extends below the mid-span loading point, after which no new shear cracks appear until the concrete in the span is crushed and destroyed. Finally, the ultimate load of the RC-control beam is 378 kN, and the ultimate mid-span deflection is 8.3 mm.

### 3.2. Experimental Results and Analysis of the Shear Capacity of FRP-Strengthened RC Beams

#### 3.2.1. Major Damage Phenomena and Processes

(1) RC beam reinforced with flax fiber sheets

**Figure 7:**
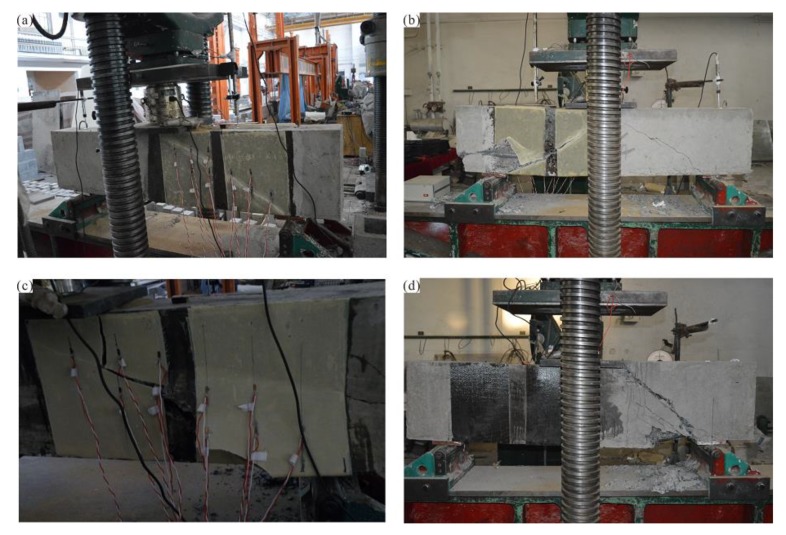
Failure mode of strengthened RC beam: (**a**) RC-FRP1, (**b**) RC-FRP3, (**c**) RC-FRP5, (**d**) RC-FRP6.

For the RC beam reinforced with flax fiber sheets, no evident shear cracks are observed on the RC surface at the beginning of loading. As the RC beam is subjected to increasing loads, the flax fiber sheets occasionally produce a slight sound, but no visible damage is observed. As the load continues to increase and the RC beam continues to emit a slight cracking noise, the flax fiber sheets near the support exhibit clear cracks in the middle and lower parts, which continue to expand along with the direction of the flax fiber sheets. As the load increases further, the flax fiber sheets suddenly began to emit a crisper sound. The load level at this time is 470–495 kN, and the mid-span deflection is 9.7–10.1 mm. Finally, along with the loud, crisp cracking sound, the flax fiber sheets near the loading point at the mid-span also detach from the beam, showing evident delamination and bulging outward. The concrete in the middle of the span is rapidly crushed, and the RC beam is broken through compression shearing. At the same time, a thin layer of concrete is attached to the inside of the flax fiber sheets. The failure mode of the RC beams strengthened with flax fiber sheets is shown in Figure 7a,b.

(2) RC beam reinforced with unidirectional basalt fiber sheets

To evaluate the effect of reinforcing RC with flax fiber sheets, basalt fiber sheet reinforcement was also employed for comparison. At the beginning of the test, no evident cracks are observed on the surface of the RC beam tension zone. As the load increases, the RC beam is placed on one side of the stirrup first with evident shear cracks, which gradually expand and deepen. When the load is increased to approximately 484 kN, the concrete beam suddenly emits a loud cracking sound, and the concrete at the bottom of the left side of the beam is peeled off. The increase in the bearing capacity of the beam is clearly slowed. When the load reaches 543 kN, the reinforced concrete beam suddenly breaks. Unlike the failure mode for the RC beams strengthened with flax fiber sheets, the basalt fiber sheets do not appear to be broken or peeled off. The shear capacity of the concrete beam reinforced with basalt fibers is close to the results for the beams reinforced with flax fiber sheets. The failure mode of the RC beam strengthened with basalt fiber sheets is shown in Figure 7d.

(3) RC beam reinforced with nano-TiO_2_ grafted flax fiber sheets

At the beginning of the test, the modified flax fiber sheets occasionally produce a slight peeling sound as the load on the concrete test beam gradually increased, but no visible damage is observed. As the load continues to increase, the concrete beam body continues to emit a slight cracking noise, and the flax fiber sheets near the support exhibit clear cracks in the middle and lower parts, which continue to expand along with the direction of the nano-TiO_2_ grafted flax fiber sheets. When the load exceeds the ultimate load of the RC beams strengthened with ordinary flax fiber sheets, the modified flax fiber sheets still have no observable fractures, indicating that the shear capacity of the beam will exceed that of the other RC beams. When the load reaches 583 kN, the flax fiber sheets near the support suddenly break in the middle and lower parts of the beam, and the concrete in the compression zone is gradually crushed. When the load reaches 632 kN, the flax fiber sheets near the mid-loading point are broken and damaged; the position is in the middle and upper part of the beam, and a thin layer of concrete is adhered to the inside. Subsequently, the side of the concrete exhibited evident shear cracks that rapidly developed from the support to the mid-span loading point. When the load reaches 653 kN, the concrete releases a huge amount of energy, debris is expelled, and the RC beam undergoes shear damage. The failure mode of the RC beam strengthened with nano-TiO_2_ grafted flax fiber sheets is shown in Figure 7c.

#### 3.2.2. Characteristic Loads and Load–Span Displacement Curves

The characteristic loads of the RC beams are listed in Table 7. Because concrete cracks in the tension zone cannot be observed after reinforcement with the flax fiber sheets, the cracking load of the concrete is not listed in this table.

In Table 7, *P*_u_ is the maximum load that the RC beam is subjected to when it is destroyed, i.e., the ultimate load of the beam; *P*_1_ and *P*_2_ are the loads at which the first and second flax fiber sheets of the RC beam detach from the beam, respectively; *δ* and *δ*_u_ are the measured deflection and ultimate deflection of the RC beam, respectively; Δ*P*_u_ is percentage increase in the shear capacity of the RC beam after reinforcement with flax fiber sheets.

The ultimate load of the beam without reinforcement is 378 kN, and the mid-span deflection is 8.3 mm. When the RC beam is reinforced with flax fiber sheets, the bearing capacity is improved considerably. After reinforcement with ordinary flax fiber sheets, the bearing capacity of the beam reaches 559–565 kN, which is 47.8–49.6% higher than that of the unreinforced beam. The shear capacity of the beam strengthened with three layers of ordinary flax fiber sheets in the warp direction is 565 kN, and the mid-span ultimate deflection is 12.2 mm. When six layers of latitudinal ordinary flax fiber sheets are used to reinforce the beam, the shear capacity is 559 kN, and the mid-span deflection is 11.8 mm. When reinforced with one layer of unidirectional basalt fiber, the shear capacity of the RC beam is 543 kN, and the mid-span ultimate deflection is 14.6 mm. The shear reinforcement effect of the basalt fiber sheets on the reinforced concrete beam is similar to that of the ordinary flax fiber sheets. When the beam is reinforced with nano-TiO_2_ grafted flax fiber sheets, the shear capacity is considerably improved to 653 kN, which is 172.8% that of the unreinforced RC beam, and thus a considerable reinforcement effect is achieved. At the same time, the beam mid-range deflection reached 14.6 mm. This indicates that the flax fiber sheets can improve the ductility of the RC beam, allowing the beam to undergo a slower load growth process, and thus the stress redistribution process inside the beam can be fully carried out.

**Figure 8:**
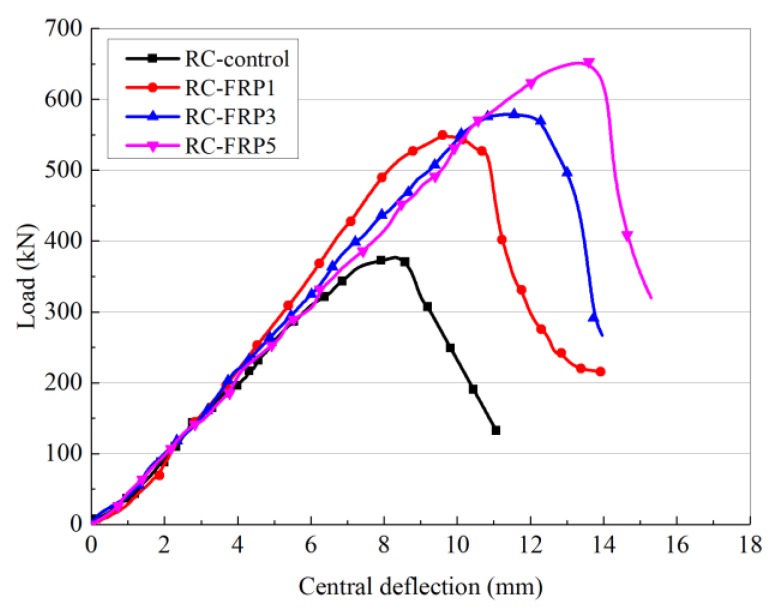
Load curves of strengthened RC beam.

The load–span displacement curves of RC beams strengthened with flax fiber sheets and nano-TiO_2_ grafted flax fiber sheets are shown in Figure 8. The results show that the flax fiber sheets can considerably improve the shear capacity of the RC beam, and the development trends of the load–span displacement curves are relatively similar under different reinforcement schemes. When the load is small, the mid-span displacement of the RC beam increases gradually. When the flax fiber sheets are subjected to an increasingly large external force, the growth of the mid-span displacement of the concrete gradually slows, indicating that a stress redistribution process is occurring inside the concrete. When the RC beam was reinforced with nano-TiO_2_ grafted flax fiber sheets, the shear capacity of the beams reached 653 kN, which is the maximum value for all of the beams tested. The span-to-medium limit deflection of the concrete beam reached 14.6 mm, which is less than when it is not strengthened, but represents a considerable increase of approximately 6.3 mm compared with the unreinforced beam.

The test results show that, on the one hand, flax fiber sheets can effectively enhance the shear resistance of RC beams and improve the shear capacity of concrete beams. The effectiveness of the structural reinforcement has been verified. On the other hand, reinforcement with flax fiber sheets also improves the ductility of the concrete beam, and the resistance of the concrete beam to deformation is enhanced. Therefore, RC beams reinforced with flax fiber sheets not only meet the bearing capacity requirements, but they also meet the structural ductility requirements.

#### 3.2.3. Strain Distribution in the Flax Fiber Sheets

**Figure 9:**
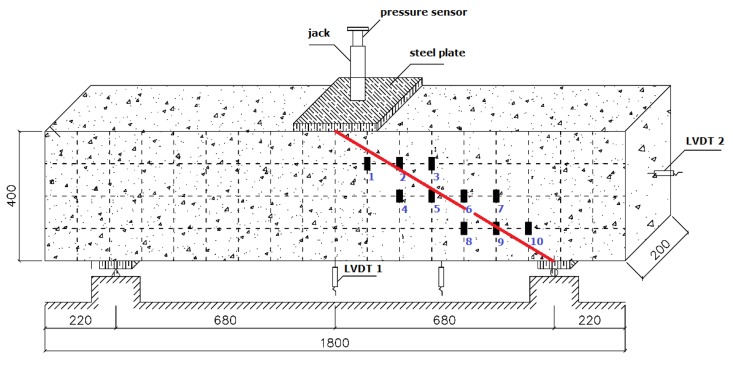
Strain gauges on the RC-control beam (unit: mm).

For comparison, 10 sets of resistance strain gauges were placed on the right side of the unplaced stirrups; the strain gauge locations and numbers are shown in Figure 9. All of the resistance strain gauges were attached to both sides of the main shear crack, which can accurately detect the strain change generated when the concrete beam cracks.

Taking the mid-plane of the concrete beam as the reference plane, the 10 resistance strain gauges are divided into three groups: the first group (numbered 1, 2, and 3), the second group (numbered 4, 5, 6, and 7), and the third group (numbered 8, 9, and 10). Each set of resistance strain gauges recorded data for loads of 50 kN (13.2%), 150 kN (39.7%), 220 kN (58.2%), 300 kN (79.4%), and 350 kN (92.6%) as representative measurement points. The strain distribution of each resistance strain gauge is shown in Figure 9 (the abscissa number in the figure corresponds to the resistance strain gauge number).

**Figure 10:**
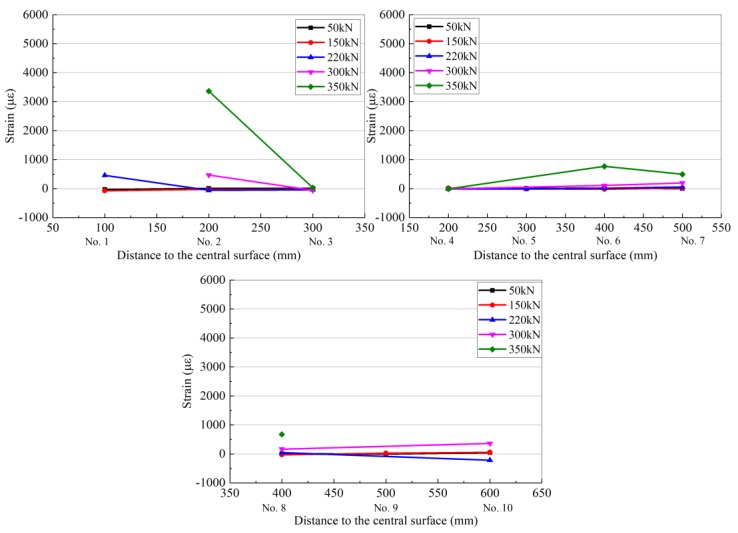
Strain distribution of concrete for RC-control beam.

Table 8 and Figure 10 shows that as the load level increases gradually, some of the resistance strain gauges become non-operational owing to excessive deformation; thus, some data points in the figure are missing. The shear crack in the ordinary reinforced concrete beam gradually develops from the support obliquely upward. With a gradual increase in the load, the direction of the main shear crack may appear as in Figure 7, and a major shear crack is formed from several shear cracks, called a critical shear crack.

The curve in Figure 10 shows that the development of the shear crack in the ordinary RC beam passes through resistance strain gauge numbers 8, 6, and 2. After the critical shear crack occurs, the beam can continue to bear the load until the stirrup intersecting the shear crack reaches the yield strength and the concrete in the shear zone is crushed. Meanwhile, the concrete at the uppermost shearing zone of the shear crack reaches the ultimate strength under the combined action of shear stress and compressive stress, and the steel and concrete are fully utilized, similar to the normal section failure of the reinforced beam. However, compared with the normal section failure of the beam, the damage is sudden and is a brittle failure. As the shear span ratios of the reinforced concrete beams prepared in this study are close to 2, the damage to the reinforced concrete beams is a typical shear failure.

**Figure 11:**
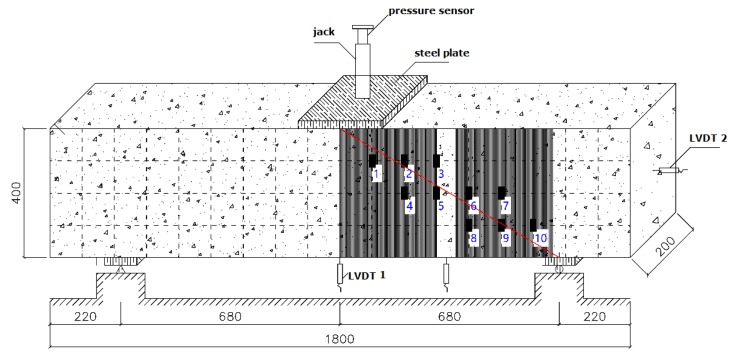
Strain gauges on the surface of flax fiber sheet (unit: mm).

Unlike the ordinary RC beam, after reinforcement with flax fiber sheets, most of the surface of the concrete beam is covered and does allow for determining the strain of the concrete. Therefore, in this study, the same 10 resistance strain gauges are attached to the surface of the flax fiber sheets on each RC beam to measure the strain distribution of the flax fiber sheets. The locations and numbers of the resistance strain gauges on the RC beams are shown in Figure 11.

All of the resistance strain gauges are attached to both sides of the main shear crack, which can accurately detect the cracking of the RC beam and cause a change in the strain of the flax fiber sheets. Similar to the strain distribution on the concrete surface, when calculating the strain distribution of the flax fiber sheets, the 10 resistance strain gauges are divided into three groups: the first group (numbered 1, 2, and 3), the second group (numbered 4, 5, 6, and 7), and the third group (numbered 8, 9, and 10). At the same time, the resistance values measured by the strain gauges are recorded as the load is gradually increased.

**Figure 12:**
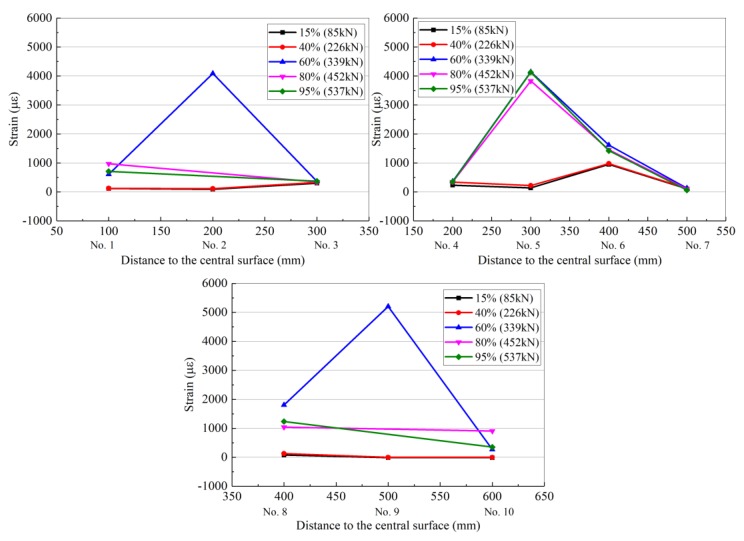
Strain distribution of flax fiber sheet for the RC-FRP1.

**Figure 13:**
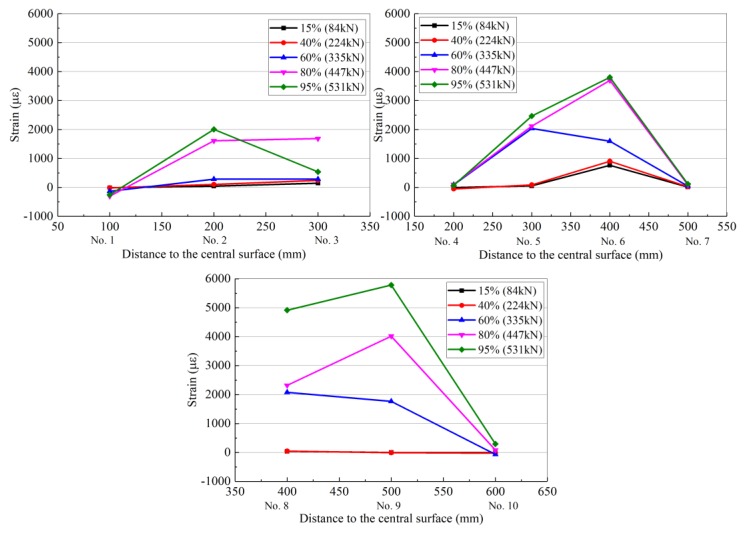
Strain distribution of flax fiber sheet for the RC-FRP3.

**Figure 14:**
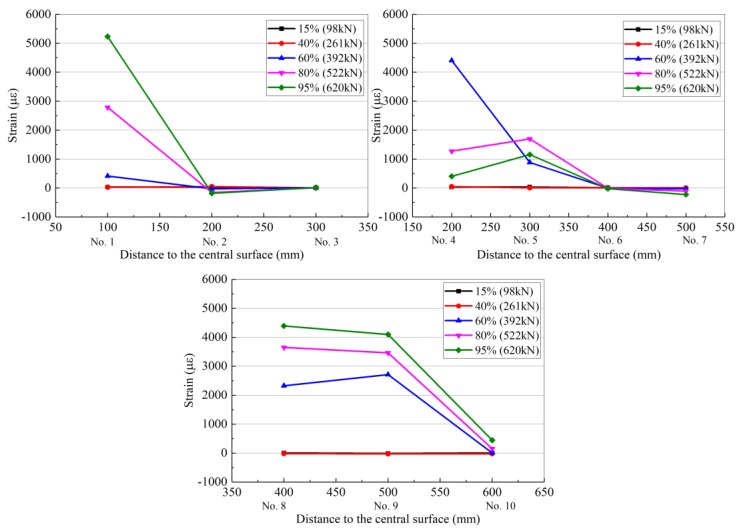
Strain distribution of flax fiber sheet for the RC-FRP5.

Each set of resistance strain gauges (1–10 in Figure 11) collected data at loads of 15%, 40%, 60%, 80%, and 95% of the ultimate load as representative measurement points, which indicate the degree of load. The strain distribution of each resistance strain gauge is shown in Table 9 and Figure 12, Figure 13 and Figure 14 for RC-FRP1, RC-FRP3, and RC-FRP5, respectively (the abscissa number in the figure corresponds to the resistance strain gauge number).

As can be seen in Table 9 and Figure 12, Figure 13 and Figure 14, when the load is low, i.e., before cracking of the RC beam occurs, the strain of the fiber sheets is small, which indicates that the flax fiber sheets have not yet assumed a sufficient external force (15% stress level and 40% stress level curves in Figure 12, Figure 13 and Figure 14). As the load level gradually increases, the RC beam gradually approaches the cracking load, and the strain of the flax fiber sheets suddenly increases, indicating that the flax fiber sheets attached to the side of the concrete beam begin to function (the 60% horizontal curve in Figure 12, Figure 13 and Figure 14). Different reinforcement layouts could show a different trend development of strain distribution of flax fiber sheet. In Table 8 and Table 9, the collected data at loads of 95% of the ultimate load, the strain of RC-control beam (No.2 strain gauge) is almost 3368.4 µε. At the same level, the strain of RC-FRP1, RC-FRP3, RC-FRP5 are lost, 2005.5 µε and −181.8 µε, respective. This shows that, with respect to strain distribution, the reinforcement method has a great influence and should be judged in the later application.

At this stage, the flax fiber sheets exert the same effect as the stirrup on the other side. Thus, as the load is gradually increased, the load on the flax fiber sheets is gradually increased, and the generated strain is also gradually increased.

As shown in Figure 10, the main shear crack in the RC beam passes through the strain gauge numbers 9, 6, 5, and 2 in sequence. Therefore, as the load gradually increases, these resistance strain gauges are gradually withdrawn from the working state. The cracking of the concrete shear section causes the concrete to effectively withdraw from performing work, and thus its contribution to the shear performance is low. At this time, the shear capacity is mainly provided by the flax fiber sheets, and the flax fiber sheets exhibit the largest strain in the middle of the beam.

As can be seen from Figure 12, Figure 13 and Figure 14, the flax fiber sheets near the crack have greater strains, while the strains farther away from the crack are smaller. This is primarily because the peeling of the flax fiber sheets gradually extended from the crack to the upper and lower free ends. At the same time, the failure mode of the RC beams strengthened with flax fiber sheets indicates that the fracture failure of the flax fiber sheets occurs before the peeling damage to the flax fiber sheets and concrete interface, which is due to the low tensile strength of the flax fiber sheets. According to previous research results [24], the effect of a fiber cloth attached in the longitudinal direction of a concrete beam on the shear capacity of the beam is not evident [24]. However, flax fiber sheets attached to the side of the concrete beam at other angles can improve the shear capacity of the concrete beam. Therefore, the arrangement of the primary force direction of the flax fiber sheets should form a certain angle with the shear crack in the reinforced concrete. This arrangement is beneficial for the performance of the flax fiber sheets, and it has strong engineering feasibility and economic rationality.

### 3.3. Shear Capacity Model of RC Beams Reinforced with Flax Fiber Sheets

Because the RC beam designed in this study has a one-side arrangement of stirrups, the damage of RC-FRP 1, RC-FRP-3 and RC-FRP-5 occurs on the side without the vertical steel bars. This is in agreement with previous research results [25,26,27]. The force equation for an RC beam without web steel bars, which considers the influence of the vertical steel bars on the shear behavior of the RC beam is shown in Equation (1) below [28].

*τ*/*f_c_*′ = *Aσ*/*f_c_*′ + *B* *σbξh*_0_ = *ρ_s_bh*_0_*f_s_* *V_c_* = *τbξh*_0_ *V_c_d_v_* = *σbξh*_0_(*h*_0_ − *ξh*_0_/2) (1)

In Equation (1),

*τ*—shear stress of the reinforced concrete (MPa);
*f_c_*′—compressive strength of the concrete (MPa);
*σ*—normal stress of the reinforced concrete (MPa);
*b*—width of the reinforced concrete beam (mm);
*h*_0_—effective height of the section of RC beam (mm);
*ξh*_0_—ultimate height of compression zone of RC beam (mm);
*ρ_s_*—steel bars ratio of the reinforced concrete beam;
*f_s_*—stress of the vertical steel bars under extreme conditions (MPa);
*V_c_*—contribution of the vertical steel bars in the concrete to the shear bearing capacity (kN);
*d_v_*—axial distance from the mid-load point to the support point (mm);
*A*, *B*—calculation parameters for the shear capacity of RC beams without web steel bars.

By solving for the four unknowns in Equation (1), a unified calculation formula for the shear capacity of the RC beams without web steel bars can be obtained, as shown in Equation (2).

*V_f_* = *φf_c_*′*bh*_0_ *Φ* = (2*B* + A*ρ_s_f*_s_/*f_c_*′)/(2*Bλf_c_*′/*ρ_s_f_s_* + 1) *A* = 0.837, *B* = 1.075 (2)

In Equation (2),

*V_f_*—contribution of the flax fiber sheets to the shear bearing capacity (kN).

Substituting the test results from this study into Equation (1), the shear span ratio is *λ* = *d_v_*/*h*_0_ = 680/335 = 2.03, RC web width (*b*) is 200 mm, steel bars ratio of RC beams (*ρ_s_*) is 0.0368, and thus *V_c_* is calculated to be 437.5 kN.

Using Equation (2) in the current national Standard for the Construction Specification for Concrete Structures (GB 50367-2013) [28], the contribution of the shear capacity of ordinary flax fiber sheets and flax fiber sheets grafted with nano-TiO_2_ to the RC beams can be calculated. When reinforced with ordinary flax fiber sheets, the flax fiber cloth in the warp and weft directions contribute 33.9 kN and 43.7 kN to the shear capacity of RC beams, respectively. When using flax fiber sheets grafted with nano-TiO_2_, the contribution of the flax fiber sheets to the shear capacity of RC beams is 47.3 kN. Moreover, the shear capacity of the RC beams is added to the shear capacity contributed by the ordinary and nano-TiO_2_ grafted flax fiber sheets. A comparison of calculated shear capacity results for the RC beams is given in Table 10.

After reinforcement with flax fiber sheets, the model calculation results for the shear capacity of RC beams are smaller than the test results, and the error can reach −25.8%. This is because China’s national regulations use the ultimate tensile strength design value of the flax fiber sheets as the design indicator. When the more common structural reinforcement materials, such as carbon fiber sheets and glass fiber sheets, are used for shear reinforcement, the stress–strain curve is approximately linear. However, the flax fiber sheets have evident nonlinear properties. When the tensile strength of the flax fiber reaches the limit value, the strain can still grow slowly, and the elongation at breaking is considerably larger than that of carbon fiber sheets, by a factor of approximately 4. Therefore, using the ultimate tensile strength as a control index for the calculation of the shear capacity of RC beams strengthened with flax fiber sheets is clearly unsuitable and inaccurate.

Based on the above analysis, this study proposes to use the ultimate strain of the flax fiber sheets as the control index, and it derives an equation for calculating the shear capacity of RC beams strengthened with flax fiber sheets and flax fiber sheets grafted with nano-TiO_2_. Analysis of the existing models and research results indicates that the shear capacity of RC beams is closely related to the cuff ratio (*E_f_t_f_*) of flax fiber sheets [24]. Therefore, Equation (2) is modified to yield Equation (3).

*V_f_* = 0.9*ε_f_E_f_ρ_f_bh*_0_(1 + cot*β*)sin*β* (3)

In Equation (3),

*E_f_*—elastic modulus of the FRP composites (GPa);
*ρ_f_*—clamping ratio of the FRP composites;
*ε_f_*—effective strain of the FRP composites (mm/mm).
*β*—angle between the flax fiber sheets and the longitudinal axis of the RC beam (°).

For the two methods of full package pasting and U-type pasting, the relevant equation is as follows:

(4) $$\varepsilon_{f}=\{\begin{array}{c} 0.17\frac{f_{c}^{2/3}}{E_{f}\rho_{f}}\varepsilon_{fu}\\ \min\{\begin{array}{c} 0.65\frac{f_{c}^{2/3}}{1000E_{f}\rho_{f}}\times 10^{-3}\\ 0.17{\left(\frac{f_{c}^{2/3}}{1000E_{f}\rho_{f}}\right)}^{0.3}\varepsilon_{fu} \end{array} \end{array}$$

In Equation (4),

*f_c_*—concrete compressive strength design value (MPa).

(5) $$V_{f}=0.9\varepsilon_{f}E_{f}\frac{2t_{f}\omega_{f}}{b_{c}s_{f}}bh_{0}\left(1+\cot\beta \right)\sin\beta \to V_{f}=0.9\varepsilon_{f}\frac{2\omega_{f}}{bs_{f}}bh_{0}\left(1+\cot\beta \right)\sin\beta \left(E_{f}t_{f}\right)$$

In Equation (5),

*E_f_*—tensile modulus of the flax fiber sheets (GPa);
*t_f_*—thickness of the flax fiber sheets (mm);
*ε_f_*—effective strain of the flax fiber sheets (mm/mm);
*w_f_*—width of the flax fiber sheets (mm);
*s_f_*—spacing between the center points of adjacent flax fiber sheets (mm).

Defining parameter *B*(*ε_f_*) = 0.9*B*(*ε_f_*) × 2*w_f_*/*bs_f_* × *bh*_0_(1 + cot*β*)sin*β* converts Equation (5) to the following:

*V_f_* = *B*(*ε_f_*)(*E_f_t_f_*) (6)

In Equation (6),

*B*(*ε_f_*)—parameter related to the ultimate strain of the flax fiber sheets.

Collating the mechanical properties of the flax fiber composites and research results for the shear properties of RC beams, the correlation curve between *V_f,exp_*/*B* and *E_f_t_f_* can be obtained as shown below. As indicated, *V_f,exp_*/*B* and *E_f_t_f_* exhibit a good linear correlation, with correlation coefficient values reaching 0.996.

*V_f,exp_*/*B* = 0.194*E_f_t_f_* + 3.504 (*r* = 0.996) (7)

In Equation (7),

*V_f,exp_*—contribution of the flax fiber sheets and flax fiber sheets grafted with nano-TiO_2_ to the shear capacity of RC beams (kN).

Defining parameters *k* = 0.194 and *C* = 3.504, the contribution of flax fiber sheets and flax fiber sheets grafted with nano-TiO_2_ to the shear capacity of RC beams is obtained. The formula is as follows:

(8) $$V_{f,exp}=\left[k\left(E_{f}t_{f}\right)+C\right]B=\left[k\left(E_{f}t_{f}\right)+C\right]0.9\varepsilon_{f}\frac{2\omega_{f}}{bs_{f}}bh_{0}\left(1+\cot\beta \right)\sin\beta$$

In Equation (8),

*k*, *C*—curve fitting parameters.

In this calculation, the influence of the vertical steel bars of the RC beams on the shear bearing capacity is considered. Therefore, the equations for the shear capacity of RC beams strengthened with flax fiber sheets and flax fiber sheets grafted with nano-TiO_2_ are obtained by adding the Equation (1) and Equation (8), as follows:

(9) $$V_{exp}=V_{c}+V_{f}=\frac{2B+A\frac{\rho_{s}f_{s}}{f_{c}^{‘}}}{2B\lambda \frac{f_{c}^{\prime }}{\rho_{s}f_{s}}+1}f_{c}^{\prime }bh_{0}+\left[k\left(E_{f}t_{f}\right)+C\right]0.9\varepsilon_{f}\frac{2\omega_{f}}{bs_{f}}bh_{0}\left(1+\cot\beta \right)\sin\beta$$

In Equation (9),

*V_exp_*—shear capacity of the RC beam (kN);
*λ*—shear span ratio of the RC beam, *λ* = *d_v_*/*h*_0_.

The experimental results for the shear capacity of reinforced concrete beams reinforced with ordinary flax fiber sheets and flax fiber sheets grafted with nano-TiO_2_ are compared with the calculation results of the proposed model in Table 11. The results of the shear capacity tests are in good agreement with the calculation results for the shear capacity of RC beams strengthened with flax fiber sheets and flax fiber sheets grafted with nano-TiO_2_. This indicates that the ultimate strain of flax fiber sheets is suitable and accurate as a control index.

The shear capacity of the RC beams after reinforcement exhibits a good linear relationship with the cuff ratio of the flax fiber sheets. According to the Standard for the Construction Specification for Concrete Structures (GB 50367-2013) [28], the error between the calculation results and test results of shear capacity of RC beams strengthened with ordinary flax fiber sheets and flax fiber sheets grafted with nano-TiO_2_ was very small, which is −1.34% to 1.94%.

For the calculation of shear capacity of RC beams strengthened with the flax fiber sheet, the calculation result is in good agreement with the test result, furthermore, it is more safe. In view of the limited number of specimens in this paper, further bases for the design and calculation of this strengthened RC beam will be obtained through further tests.

## 4. Conclusions

Based on previous research on the properties of flax fiber composites, flax fiber sheets were used to improve the shear resistance of RC beams. The bearing characteristics and failure modes of RC beams strengthened with flax fiber sheets were investigated, and the main conclusions of this study are as follows:

(1) Compared with unreinforced RC beams, the shear capacity and mid-span deflection of beams strengthened with flax fiber fabric sheets is greatly improved.
(2) The reinforcing effect of flax fiber sheets grafted with nano-TiO_2_ is greater than that of unmodified flax fiber sheets. After reinforcement with ordinary flax fiber sheets, the shear capacity of RC beams is 550–579 kN, which is 45.4–53.2% higher than that of unreinforced RC beams.
(3) After reinforcement with flax fiber sheets grafted with nano-TiO_2_, the shear capacity of RC beams is greatly improved to 653 kN, which is 72.8% higher than that of unreinforced RC beams. At the same time, the mid-range deflection of the beam reached 14.6 mm, which is 75.9% higher than that of unreinforced RC beams.
(4) Taking the ultimate strain of the flax fiber sheets as a control index, the shear capacity of the RC beams after reinforcement exhibits a good linear relationship with the cuff ratio of the flax fiber sheets. An equation for calculating the shear capacity of RC beams strengthened with ordinary flax fiber sheets and flax fiber sheets grafted with nano-TiO_2_ was preliminarily proposed through curve fitting.
(5) For the calculation of shear capacity of RC beams strengthened with the flax fiber sheet, the calculation result is in good agreement with the test result. Furthermore, it is safer. In view of the limited number of specimens in this paper, further bases for the design and calculation of this strengthened RC beam will be obtained through further tests.

## Figures and Tables

**Table 1:** Mechanical properties of the steel bars in the RC beams.

Rebar Type	Grade	Diameter (mm)	Yield Strength (MPa)	Tensile Strength (MPa)	Elastic Modulus (GPa)	Elongation (%)
Vertical steel bars	HRB400	25	410	570	200	20.0
Structural steel bars	HRB400	14	410	565	200	21.5
Erecting steel bars	HRB400	12	390	560	200	20.0
Stirrup	HPB300	8	310	450	210	25.0

**Table 2:** Technical parameters of the nano-TiO_2_.

Crystal Phase	Average Particle Size (nm)	Purity (%)	Specific Surface Area (m^2^/g)	Bulk Density (g/cm^3^)
Anatase phase	10 ± 5	>99	200 ± 30	0.25–0.35

**Table 3:** Technical parameters of the flax fiber sheets.

Graft Content (%)	Molding	Tensile Strength (MPa)		Tensile Modulus (GPa)		Inter-Laminar Shear Strength (MPa)
		Radial	Latitudinal	Radial	Latitudinal	
2.34	Hand lay-up	297.4	225.6	6.83	6.61	48

**Table 4:** Technical parameters of the unidirectional basalt fiber sheets.

Single Fiber Diameter (µm)	Tensile Strength (MPa)	Elastic Modulus (GPa)	Elongation (%)	Areal Density (g/m^2^)	Nominal Thickness (mm)
13	2100	105	2.1	800	0.4

**Table 5:** Experimental instruments and equipment used to test the shear capacity of RC beams.

Test Instrument and Equipment	Models	Manufacturer
High-speed static strain test analysis system	16 aisle, DH3820	Jiangsu Donghua Testing Technology Co., Ltd. (Jingjiang, China)
Pressure sensor	250 t, GD-250T	Fuzhou Jingkong Automation Equipment Co., Ltd. (Fuzhou, China)
Linear variable differential transformer (LVDT)	25 mm, MHR-25	Shanghai Ruihong Testing Technology Co., Ltd. (Shanghai, China)
Resistance strain gauges	120 Ω, BE120-10AA 120 Ω, BQ120-60AA	AVIC Electric Instrument Co., Ltd. (Beijing, China)

**Table 6:** Configuration of the reinforcement with fiber sheets.

RC Beam	Fiber Sheets Type	Main Direction	Paste Layers
RC-FRP1	Ordinary flax fiber sheets	Radial	3
RC-FRP3	Ordinary flax fiber sheets	Latitudinal	6
RC-FRP5	Flax fiber sheets grafted with nano-TiO_2_	Radial	3
RC-FRP6	Unidirectional basalt fiber sheets	-	1

Note: RC-FRP1 and RC-FRP3: RC beam strengthened by 3 and 6 layers of ordinary flax fiber sheets. RC-FRP5: RC beam strengthened by 3 layers of flax fiber sheets grafted with nano-TiO_2_. RC-FRP6: RC beam strengthened by 1 layer of basalt fiber sheets.

**Table 7:** Characteristics load of the RC beams.

Beam	*P*_1_ (kN)	*δ*_1_ (mm)	*P*_2_ (kN)	*δ*_2_ (mm)	*P*_u_ (kN)	*δ*_u_ (mm)	*P*_1_/*P*_u_ (%)	*P*_2_/*P*_u_ (%)	Δ*P*_u_ (%)
RC-control	--	--	--	--	378	8.3	--	--	--
RC-FRP1	488	10.1	528	11.2	565	12.2	86.2	93.3	49.6
RC-FRP3	473	10.0	535	11.2	559	11.8	84.7	95.8	47.8
RC-FRP5	583	13.4	632	14.3	653	14.6	89.2	96.8	72.8
RC-FRP6	--	--	--	--	543	11.7	--	--	43.5

**Table 8:** Strain distribution of concrete for RC-control beam.

Beam	Resistance Strain Gauge	Strain Distribution				
		15%	40%	60%	80%	95%
RC-control	No.1	−29.1	−74.1	459.9	•	•
	No.2	9.31	−22.5	−61.2	468.5	3368.4
	No.3	10.8	−26.1	−43.1	−53.7	29.9
	No.4	−1.1	16.9	−13.2	−7.4	−7.6
	No.5	8.4	−9.2	−12.7	•	•
	No.6	16.2	−18.0	12.5	108.5	768.7
	No.7	−0.4	15.5	56.5	198.8	493.4
	No.8	6.5	−28.3	42.1	165.3	676.0
	No.9	−1.9	21.6	•	•	•
	No.10	39.7	58.4	−217.7	361.4	•

Note: •: the loss of data due to the fact that the resistance strain gauge has fallen off the concrete surface.

**Table 9:** Strain distribution of flax fiber sheet for the RC-FRP1, RC-FRP3 and RC-FRP5.

Beam	Resistance Strain Gauge	Strain Distribution				
		15%	40%	60%	80%	95%
RC-FRP1	No.1	115.2	127.6	606.1	971.1	710.0
	No.2	94.6	111.8	4087.0	•	•
	No.3	301.0	327.0	364.1	347.4	367.2
	No.4	234.1	337.1	363.3	349.6	355.4
	No.5	140.9	221.2	4142.2	3830.4	4131.1
	No.6	947.5	982.5	1620.0	1457.4	1420.8
	No.7	109.2	116.4	128.2	102.8	61.9
	No.8	74.1	125.4	1803.1	1040.7	1232.9
	No.9	−14.0	−3.1	5204.4	•	•
	No.10	−12.0	−5.9	263.6	904.3	352.4
RC-FRP3	No.1	−8.6	−15.2	−131.2	−305.5	−258.7
	No.2	46.5	101.1	288.0	1611.2	2005.5
	No.3	147.5	244.4	286.1	1687.4	539.9
	No.4	−3.8	−48.0	95.5	101.2	70.4
	No.5	53.9	85.5	2037.7	2116.6	2465.8
	No.6	764.4	906.2	1596.5	3695.2	3803.6
	No.7	13.7	21.2	46.5	85.0	115.2
	No.8	41.1	48.8	2081.9	2317.5	4917.1
	No.9	0	−4.1	1764.7	4020.1	5782.1
	No.10	−3.3	−21.0	−70.4	85.7	297.0
RC-FRP5	No.1	32.9	26.3	415.3	2789.1	5234.7
	No.2	33.2	50.7	−24.7	−154.7	−181.8
	No.3	7.7	6.1	11.9	10.2	19.2
	No.4	35.8	50.9	4408.5	1271.5	403.4
	No.5	35.8	9.2	882.3	1696.4	1156.1
	No.6	12.7	9.9	−7.1	−10.1	−17.8
	No.7	−3.4	3.5	−22.8	−110.6	−227.5
	No.8	5.5	−18.9	2325.6	3650.9	4392.5
	No.9	−12.2	−26.7	2714.0	3464.9	4096.2
	No.10	3.9	−22.2	4.2	147.4	443.6

Note: • the loss of data due to the fact that the resistance strain gauge has fallen off the concrete surface.

**Table 10:** Experimental and standard calculation results for the shear capacity of RC beams.

Beam	Shear Bearing Capacity Test Results (kN)	Shear Bearing Capacity Specification Formula Calculation Results (kN)	Error (%)
RC-control	378	437.5	15.7
RC-FRP1	565	471.4	−16.6
RC-FRP3	559	481.2	−13.9
RC-FRP5	653	484.8	−25.8

**Table 11:** Calculation results of shear capacity model for the RC beam strengthened by flax fiber sheets.

Beam	Shear Bearing Capacity Test Results (kN)	Shear Bearing Capacity Model Calculation Results (kN)	Error (%)
RC-FRP1	565	557.4	−1.34
RC-FRP3	559	569.9	1.94
RC-FRP5	653	646.3	−1.02
